# Meta-analysis of structural MRI studies in anorexia nervosa and the role of recovery: a systematic review protocol

**DOI:** 10.1186/s13643-021-01799-y

**Published:** 2021-09-13

**Authors:** Melissa J Dreier, Avery L. Van De Water, Danielle L. Kahn, Kendra R. Becker, Kamryn T. Eddy, Jennifer J. Thomas, Laura M. Holsen, Elizabeth A. Lawson, Madhusmita Misra, Amanda E. Lyall, Lauren Breithaupt

**Affiliations:** 1 Eating Disorders Clinical and Research Program, Massachusetts General Hospital, Boston, MA USA; 2 Present Address: Department of Psychology, Rutgers University, Piscataway, NJ USA; 3 Neuroendocrine Unit, Department of Medicine, Massachusetts General Hospital and Harvard Medical School, Boston, MA USA; 4 Department of Psychiatry and Division of Women’s Health, Department of Medicine, Brigham and Women’s Hospital, Boston, MA USA; 5 Present Address: Department of Neuroscience, University of Iowa, Iowa City, IA USA; 6 Present Address: Ferkauf Graduate School of Psychology, Yeshiva University, Bronx, NY USA; 7 Department of Medicine, Harvard Medical School, Boston, MA USA; 8 Department of Psychiatry, Harvard Medical School, Boston, MA USA; 9 Division of Pediatric Endocrinology, Department of Pediatrics, Harvard Medical School, Boston, MA 02114 USA

**Keywords:** Anorexia nervosa, Structural MRI, Meta-analysis, Seed-based d mapping, Recovery

## Abstract

**Background:**

Anorexia nervosa (AN) is associated with structural brain abnormalities. Studies have reported less cerebral tissue and more cerebrospinal fluid (CSF) in individuals with AN relative to healthy controls, although findings are variable and inconsistent due to variations in sample size, age, and disease state (e.g., active AN, weight-recovered AN). Further, it remains unclear if structural brain abnormalities observed in AN are a consequence of specific brain pathologies or malnutrition, as very few longitudinal neuroimaging studies in AN have been completed.

**Methods:**

To overcome this issue, this comprehensive meta-analysis will combine region-of-interest (ROI) and voxel-based morphometry (VBM) approaches to understand how regional and global structural brain abnormalities differ among individuals with AN and healthy controls (HCs). Additionally, we aim to understand how clinical characteristics and physiological changes during the course of illness, including acute illness vs. weight recovery, may moderate these structural abnormalities. We will create an online database of studies that have investigated structural brain abnormalities in AN. Data will be reviewed independently by two members of our team using MEDLINE databases, Web of Science, PsycINFO, EMBASE, and CINAHL. We will conduct ROI and VBM meta-analysis using seed-based d mapping in AN and HCs. We will include all studies that include structural neuroimaging of individuals with AN (both acute and weight-recovered) and HCs between January 1997 and 2020.

**Discussion:**

This systematic review will assess the effects of AN compared to HC on brain structure. Futhermore, it will explore the role of acute AN and weight-recovered AN on brain structure. Findings will help researchers and clinicians to better understand the course of illness in AN and the nature of recovery, in terms of weight, malnutrition, and the state of the brain.

**Systematic review registration:**

PROSPERO CRD42020180921

**Supplementary Information:**

The online version contains supplementary material available at 10.1186/s13643-021-01799-y.

## Background

Anorexia nervosa (AN) is a severe and lethal psychiatric disorder, with a mortality rate of 10% [1–5]. Although the pathoetiology of the disorder remains uknown, genetic, environmental, and neurobiological factors have all been shown to contribute [6–9]. Structural brain alterations in AN have been explored for three decades, with the first published study in 1990 [10]. However, it remains unclear whether the structural brain abnormalities reported in AN are a consequence of starvation or an indication of an underlying biological mechanism preceding AN symptomatology. Research suggests that these inconsistencies are the result of small samples and/or heterogeneous patient samples and neuroadiological study quality [11, 12]. A meta-analysis of all published studies can clarify cortical grey matter abnormalities in AN in relation to the course of disease and recovery in this complex illness.

Previous meta-analyses investigating structural brain changes in AN from 2010 to 2018 demonstrate that individuals with AN have lower grey [13–16] and white [13, 14, 16] matter volumes compared to healthy controls, affecting almost the whole cortex. One meta-analysis, conducted by Seitz et al. [16], included individuals who had weight-recovered from AN (i.e., individuals who had regained weight lost due to AN but who had not necessarily recovered psychologically), in comparison to healthy controls. Findings from this work demonstrate that the longer an individual is in recovery, the more grey matter restoration takes place [16]. However, it is unknown whether specific brain regions experience differential restoration as individuals recover or if recovery of gray matter is associated with symptom improvement (e.g., full recovery). Furthermore, definitions of recovery from AN differ widely among researchers and clinicians [17], and understanding which indicators (e.g., weight-recovery, remission of core behaviors or cognitions) are related to the observed structural brain abnormalities in AN requires further investigattion [18]. A more precise definition of recovery uniting neurobiological substrates with clinical outcomes would inform definitions of recovery in AN.

Presently, there are two common methods to investigate structural brain changes in AN: voxel-based morphometry (VBM) and region-of-interest (ROI). VBM is a coordinate-based approach that surveys the entire brain to examine regional changes in volume. The ROI approach focuses on specified regions-of-interest only. The small number of meta-analyses in AN to date have focused on studies using a VBM approach. VBM approaches are effective in summarizing consistently reported peaks, but tend to ignore nonsignificant data. To circumvent this challenge, new Seed-based Mapping (SDM) methods (e.g., SDM effect-size) allow for the inclusion of 3D-statistical maps to increase accuracy. To date, only one meta-analysis has used SDM-effect size in acute AN [15] and no meta-analytic studies have used this approach to investigate brain structure in weight-recovered AN. VBM studies present less bias than region-of-interest studies (ROI); however, ROI and VBM meta-analyses analyze MRI data in different ways. Excluding all ROI analyses may itself lead to a bias as a critical amount of studies may not be considered [19]. To date, none of the meta-analyses in AN have included ROI based studies and a direct comparison of ROI and VBM studies has thus not been completed.

A meta-analysis utilizing ROI and VBM image analysis to examine grey matter in individuals with actute AN and individuals who are weight-recovered will provide an opportunity to increase statistical power. This meta-analysis will differ from previous meta-analytic work, which restricted investigation to only one of these measurement approaches. For the purposes of this study, we will be looking at individuals with acute AN vs. weight-recovered AN (rather than including other definitions of recovery). This will help uncover which brain regions may be most sensitive to weight loss. We would expect regions most sensitive to weight-loss (rather than psychological symptoms) to be remitted in those with weight-recovered AN because most other medical complications of AN remit with weight restoration [20]. On the other hand, regions that are more linked to psychological disturbances associated with AN may not necessarily be remitted in those with weight-recovered AN.

### Current study

This comprehensive meta-analysis of grey matter imaging studies comparing AN to control subjects will aim to answer the following questions: (1) How do global and regional structural brain abnormalities differ between individuals with acute AN versus healthy controls (HC), weight-recovered AN versus HC, and acute AN vs. weight-recovered AN? (2) How do clinical characteristics and physiological differences moderate structural brain abnormalities in individuals with AN and weight-recovered AN? To answer these questions, we will (1) collectively analyze key findings from all structural volumetric studies comparing AN to HC using both ROI and VBM and (2) use meta-regression analyses to explore key neuroradiological, study quality, demographic, clinical, and metabolic variables to explore potential moderators of the cross-sectional effects detected. We will include sub-group analyses to compare those with AN to HC and AN to weight-recovered AN cross-sectinoally. We will also compare those with weight-recovered AN individuals to HC, to make inferences on the site and timing of cortical grey matter changes in AN.

## Methods/design

The proposed systematic review will be reported in accordance with Preferred Reporting Items for Systematic Reviews and Meta-Analysis (PRISMA), and this protocol was reported in accordance with the PRISMA guidelines for systematic review protocols (PRISMA-P). This meta-analysis has been registered in PROSPERO.

### Participants

This review will consider structural magnetic resonance imaging (MRI) studies that include participants with acute AN, weight-recovered AN, and/or healthy controls. Since AN is more prevalent in females than males [21], we will include male participants with AN if sufficient data for meta-analysis are available. Previous research has established that an adequate minimum sample size is five studies [22]. However, since many studies focus exclusively on females, we will limit our inclusion to females only if we will not have adequate power to include males.

### Types of studies

This review will consider both experimental and observational studies that report data on cross-sectional and longitudinal structural MRI in individuals with acute AN and weight-restored AN, compared to healthy controls. Studies will be included only if they were published in English. Studies will be included from 1997 to the present, because this represents the timeframe in which structural MRI has been applied to study anorexia nervosa [23, 24].

### Search strategy

We will initially identify articles using MEDLINE databases. A literature search for papers related to structural neuroimaging in anorexia nervosa will be conducted using the RISMED package in R ([25]; see supplementary materials for search code). In addition to this search strategy, the reference list of all studies selected for critical appraisal will be screened for additional studies.

### Information sources

Following our search of MEDLINE databases using R code, we will search the following databases: Web of Science, PsycINFO, EMBASE, CINAHL. We will use search terms “anorexia,” “anorexia nervosa,” “MRI,” and “structural MRI.” Finally, we will identify unpublished works via (1) searching for the terms “MRI” and “anorexia nervosa” in the ProQuest Dissertations and Theses electronic database, and (2) emailing requests for unpublished or in-press studies over prominent listservs in the clinical psychology and clinical neuroscience communities (e.g., Association for Behavioral and Cognitive Therapies). We will conduct this final search method to address the potential misrepresentation of population effect sizes due to the greater likelihood that significant (vs. nonsignificant) findings will be accepted for publication [26].

### Data collection

Two review authors will independently extract data. Discrepancies will be identified and resolved through discussion with a third reviewer. Missing data will be requested from study authors via email.

We will record the following data: (1) sample size (i.e., number of current AN patients, number of recovered AN patients, number of controls), (2) diagnostic classification system used (e.g., clinical interview, clinician evaluation), (3) participant demographics (e.g., mean age of participants in each group, race, ethnicity, income), (4) participant clinical characteristics (e.g., mean age of illness onset (if reported), type of treatment (if in treatment), duration of illness, mean score from eating disorder symptom measures given (e.g., Eating Disorder Examination), lowest reported weight, highest reported weight, and type of control group used, (5) medication information (i.e., number of patients described as drug-free and number of patients using antidepressants, mood stabilizers, anxiolytics, antihistamine, or antipsychotics, and (6) MRI acquisition (i.e., slice thickness and magnetic field strength).

### Analytic plan

We aim to expand on prior work using a VBM approach by using a technique called SDM [27]. SDM is a coordinate-based meta-analytic tool that is able to combine both T-maps and coordinates into a single analysis, thus maximizing inclusion of studies. This tool also allows for the use of meta-regression to control for moderators [28]. SDM has been applied to meta-analytic work in a variety of psychiatric disorders, including one study in acute AN [15]. We will use SDM in our work to complement our ROI meta-analyses. For studies that used VBM methodology, we will use SDM to test for convergence against the null hypothesis that reported findings follow a random spatial distribution across the brain. For studies that used an ROI approach, we will achieve further specificity by demonstrating and comparing convergence abnormality in discrete brain regions among individuals with AN, weight-recovered AN, and healthy controls. Below, we outline our specific analytic plan in relation to our research questions:

*1) How do global and regional structural brain abnormalities differ between individuals with acute AN versus healthy controls (HC), weight-recovered AN versus HC, and acute AN vs. weight-recovered AN?* We will conduct a voxel-based and regional meta-analysis to probe differences in grey matter between individuals with acute AN and HC, weight-recovered AN and HC, and acute versus weight-recovered AN. Statistical analyses will be performed using random-effects inverse-weighted variance models. Subgroup analyses will be conducted where there is sufficient data to investigate. Where statistical pooling is not possible, the findings will be presented in narrative form including tables and figures to aid in data presentation, where appropriate. We will conduct sensitivity analysis to test how robust our results are relative to variations in meta-analytic method, using effect sizes and Hedges’ *g* for each brain region in our analyses for ROI and VBM. To reduce the number of comparisons in the ROI analysis, we will focus on brain regions that are significantly different from those of control subjects in either the acute-AN or weight-recovered-AN meta-analysis.

Effect sizes will be expressed as either odds ratios (for dichotomous data), weighted, or standardized final post-intervention mean differences (for continuous data), and their 95% confidence intervals will be calculated for analysis. Heterogeneity will be assessed statistically using the standard chi-squared and *I*^2^ tests. For continuous outcome measures, we will use Hedges’ *g* ([29]; the Cohen’s effect size with correction for bias from small samples).

In the case of the VBM studies specifically, we will follow the procedure for SDM [27]. This statistical software allows for researchers to include both studies that use peak coordinates and coordinates that use Statistical Parametric Mapping (SPM) t-maps in a single meta-analysis. This technique increases accuracy of effect size maps by using specific masks for structural MRI scans. Furthermore, SDM accounts for the effect size and sign of peak coordinates and weights calculations based on inter-study variance and heterogeneity.

*2) How do clinical characteristics and physiological differences moderate structural brain abnormalities in individuals with AN and weight-recovered AN?* Meta-regression will be employed to explore the potential effects of clinical characteristics, neuroradiological, and study quality. To reduce type I errors, we will select clinical variables based on key clinical questions as well as the availability of the variables in the studies. The following moderators will be considered: duration of illness, age of onset, body mass index (BMI), amount of weight gain from treatment (longitudinal studies and cross-sectional recovered AN studies), levels of depression, shape and weight concerns, body image, restriction severity, drive for thinness, frequency of binge and purge behaviors, percentage of females, and percentage of medicated participants. To explore the effects of neuroradiological techniques, we will consider the following variables: MRI slice thickness, image smoothing level, MRI field strength (Tesla), and inclusion of motion correction.

To assess whether the findings in our analysis will be related to different presentations of extreme restrictive eating: AN binge/purge subtype and AN restricting subtype AN, we will complete a supplementary set of meta-analyses in subjects based on diagnostic categories. These subgroup analyses will be exploratory in nature and will be completed to generate hypotheses to be tested in new primary studies. We will use diagnostic criteria from the Diagnostic and Statistical Manual of Mental Disorders, 5th Edition (DSM-5). Table 1 presents the diagnostic criteria for adolescents and adults for each subtype:

**Table 1:** Anorexia nervosa subtypes

Diagnosis	Criteria
Anorexia nervosa-restricting subtype	• No binge episodes in the past 3 months • No purge episodes (i.e., self-induced vomiting or the misuse of laxatives, diuretics, or enemas) in the past 3 months • Otherwise meet criteria for AN
Anorexia nervosa-binge/purge subtype	• Recurrent binge episodes in the past 3 months AND/OR • Recurrent purge episodes in the past 3 months • Otherwise meet criteria for AN

All criteria are drawn from the DSM-5 [2]

### Assessing risk of bias

Our proposed method includes VBM and ROI analysis to reduce risk of bias. We will compare results from the VBM and ROI analysis to assess agreement, which has broader implications for neuroimaging meta-analysis of other disorders and has not been done in eating disorders. VBM and ROI results often differ for the following reasons: (1) VBM involves smoothing, which biases sensitivity to brain regions dependent on the size of the kernel, (2) ROI analyses only report on a subset of brain regions and are likely to be impacted by publication bias, (3) SDM (used for VBM studies) uses coordinate data; thus, the effect size is biased toward zero in brain regions where there are no significant clusters, and (4) VBM adjusts for total global brain volumes, whereas ROI studies use absolute volumes.

We anticipate that some studies will report measures from subgroups of patients and matched controls. For example, studies may compare short-term versus long-term recovery. We will incorporate these results in the meta-analysis as two different studies, which is consistent with other meta-analyses [14, 15]. As this will increase risk for type I error, we will use a Bonferroni correction and indicate results that survive this correction. Finally, we anticipate that studies may use different methods to diagnose eating disorders in their samples. We will record the method used for diagnosis (e.g., DSM-5 versus prior editions of the DSM and/or clinical interview, such as the Eating Disorders Examination) and include these as a subgroup analysis to examine stability of results. This is particularly important for an eating disorder meta-analysis as the criteria for anorexia nervosa has significantly changed over the years to include a broader range of individuals.

The Grading of Recommendations, Assessment, Development and Evaluation (GRADE) approach for grading the certainty of evidence will be followed to assess estimates of relative risk and a ranking of the quality of the evidence based on the risk of bias, directness, heterogeneity, precision, and risk of bias of the review results.

## Discussion

Our comprehensive meta-analysis will provide a detailed summary of structural brain abnormalities and their associations with clinical variables in AN and weight-recovered AN.

### Limitations

Findings from this eventual meta-analysis should be considered in light of some limitations. First, our findings will be limited by the fact that we are only able to include studies published in English, as we do not have study team members who are native speakers of other languages. Second, due to power considerations, we anticipate that we may not be able to examine all subgroups of interest. Nonetheless, this study will provide more precise insight into structural brain changes in AN that are most sensitive to weight loss versus those that may be more sensitive to psychological disturbances of the illnesses.

## Supplementary Information

**Additional file 1.** R code describing our search of MEDLINE databases.

## Data Availability

Although availability of data is not applicable for this manuscript, the authors have included R code that can be used to replicate the search strategy for MEDLINE databases.

## Abbreviations

AN: Anorexia nervosa
BMI: Body mass index
DSM-5: Diagnostic and Statistical Manual of Mental Disorders, 5th Edition
GRADE: Grading of Recommendations, Assessment, Development and Evaluation
MRI: Magnetic resonance imaging
PRISMA: Preferred Reporting Items for Systematic Reviews and Meta-Analysis
PRISMA-P: Preferred Reporting Items for Systematic Reviews and Meta-Analysis for Systematic Review Protocols
ROI: Region of interest
SDM: Seed-based d mapping
SPM: Statistical Parametric Mapping
VBM: Voxel-based morphometry
