# Supplementary material for: Meta-analysis of structural MRI studies in anorexia nervosa and the role of recovery: a systematic review protocol
Source: Syst Rev. 2021 Sep 13;10:247. doi: 10.1186/s13643-021-01799-y (PMC8438886; doi:10.1186/s13643-021-01799-y)
Supplement: Supplementary file 1 — Additional file 1. R code describing our search of MEDLINE databases. [file 13643_2021_1799_MOESM1_ESM.docx]

**Supplementary Materials**

R code describing our search of MEDLINE databases. Comments to explain code follow “#” symbols in **bold**.

**#-------------load packages--------------------------#**

library(RISmed) #package for searching pubmed

library(stringr) #packaged used later to replace character strings

**#-------------search code--------------------------#**

**#search for any articles containing the phrase “anorexia nervosa meta-analysis**

terms <- c("anorexia meta-analysis")

for(i in 1:length(terms)){

query_term <- terms[i]

start <- 1997 **#start search in 1997**

end <- 2020 **#end search in 2020**

res <- EUtilsSummary(query_term, type='esearch', db='pubmed', datetype='pdat',

mindate=start, maxdate=end, retmax=10000)

big <- EUtilsGet(res)

**#make data frame with key pieces, except authors**

data <- data.frame(pmid=PMID(big), year=YearPubmed(big), title=ArticleTitle(big), journal=Title(big), abstract=AbstractText(big))

**#search abstracts for terms we want: keep any articles where, in the abstract the words “MRI,” “brain,” “imaging,” “meta,” or “anorexia,” are present**

data$include=ifelse(grepl("MRI", data$abstract) | grepl("brain", data$abstract) | grepl("imaging", data$abstract) | grepl("meta", data$abstract) | grepl("anorexia", data$abstract), "include", "exclude")

**#subset data to only included articles**

data <- subset(data, include=="include")

**#sort by year**

data$year <- as.numeric(data$year)

data <- data[order(data$year, na.last=NA) , ]

**#now to get the authors**

coauthors <- data.frame(Author(big)[1])

names(coauthors)<-c("LastName", "ForeName", "Initials", "order")

coauthors$author<-str_replace_all(paste(coauthors$ForeName, "_", coauthors$LastName,

sep=""), " ", "_")

coauthors$pmid=PMID(big)[1]

coa <- data.frame(pmid=coauthors$pmid, author=coauthors$author, order=coauthors$order)

ll <- length(PMID(big))

for(j in 2:ll){

coauthors <- data.frame(Author(big)[j])

names(coauthors) <- c("LastName","ForeName","Initials","order")

coauthors$author <- str_replace_all(paste(coauthors$ForeName, "_", coauthors$LastName,

sep=""), " ","_")

coauthors$pmid=PMID(big)[j]

coa<-rbind(coa,data.frame(pmid=coauthors$pmid, author=coauthors$author,

order=coauthors$order))

}

**#tack authors onto dataset**

final_authors <- merge(coa, data, by = "pmid", incomparables = NA)

final_authors <- final_authors[order(final_authors$year, na.last=NA) , ]

**#write a CSV file with all articles extracted**

write.csv(final_authors, file=paste(query_term, "-ALL AUTHORS.csv"))

}
